# Cytokines Activate JAK–STAT Signaling Pathway in MG-63 Cells on Titanium and Zirconia

**DOI:** 10.3390/ma15165621

**Published:** 2022-08-16

**Authors:** Khaled Mukaddam, Sabrina Ruggiero, Steffen M. Berger, Dietmar Cholewa, Sebastian Kühl, Daniel Vegh, Michael Payer, Michael M. Bornstein, Farah Alhawasli, Elizaveta Fasler-Kan

**Affiliations:** 1 Department of Oral Surgery, University Center for Dental Medicine, University of Basel, Mattenstrasse 40, 4058 Basel, Switzerland; 2 Department of Dentistry and Oral Health, Division of Oral Surgery and Orthodontics, Medical University of Graz, Billrothgasse 4, 8010 Graz, Austria; 3 Department of Prosthodontics, Semmelweis University, Szentkirályi utca 47, 1088 Budapest, Hungary; 4 Department of Paediatric Surgery, Children’s Hospital, Inselspital Bern, University of Bern and Department of Biomedical Research, University of Bern, Freiburgstrasse 15, 3010 Bern, Switzerland; 5 Department of Oral Health & Medicine, University Center for Dental Medicine Basel (UZB), University of Basel, Mattenstrasse 40, 4058 Basel, Switzerland; 6 Department of Biomedicine, University of Basel, University Hospital Basel, Hebelstrasse 20, 4056 Basel, Switzerland

**Keywords:** JAK–STAT pathway, cytokine, interferon, titanium, zirconia

## Abstract

Although titanium has been traditionally used as the gold standard for dental implants, recent years have seen the widespread application of zirconia implants given their superiority with regards to reduced bacterial adhesion, inflammation and cellular-interaction in terms of bio-compatibility. The JAK–STAT signaling pathway plays an important role in bone remodeling and formation. The aim of the study was to investigate the activation of the JAK–STAT pathway through different cytokines in osteoblast-like cells (MG-63) on zirconia in comparison to titanium discs. IFN-γ induced the very strong activation of STAT1 protein, IFN-α activated both STAT1 and STAT3 molecules, IL-6 activated STAT3 and IL-4 induced the activation of STAT6 on both surfaces. The activation of STAT proteins was confirmed by western blot, immunofluorescence and flow cytometry using phospho-specific anti-STAT antibodies, which recognize only phosphorylated STAT proteins. The incubation of MG-63 cells with IFN-γ caused the upregulation of MHC class I and class II proteins when MG-63 cells were grown on zirconia and titanium discs. In sum, the present study shows that the JAK–STAT pathway is activated in MG-63 cells when they are incubated on titanium or zirconia surfaces.

## 1. Introduction

The choice of implant material and its surface properties have been identified as a vital factor in achieving optimal osseointegration and long-term success [1]. For dental implants or healing abutments, titanium has been regarded the gold standard. In recent years, zirconia has been racing to become a noticeable alternative with potential to reduce peri-implant infections. With a success rate of 98.4% after five years, zirconia implants have become a promising alternative to titanium implants [2].

The JAK–STAT (Janus tyrosine kinase-Signal Transducers and Activation of Transcription) pathway regulates cell progression, organ development, cell differentiation, cell survival, cell cycle, and cell death. Understanding the effect of material choice on the JAK/STAT pathway is essential due to its importance in bone remodeling and formation, as well as its immunological significance [3].

STATs are a class of transcription factors that are activated following tyrosine phosphorylation. This cytoplasmic protein family is involved in signaling and transcription factor regulation, participating in the normal cellular responses to cytokines and growth factors. Signaling pathways are activated through cell membrane receptors, which are triggered by various ligands [4]. When the ligands are bound to the transmembrane receptors, it results in the rapid phosphorylation and activation of members of the intracellular JAK protein family. The activated receptor–kinase complexes recruit the members of the STAT protein family, which then translocate to the nucleus in a phosphorylated and dimerized form, promoting the transcription of specific genes [4]. The JAK–STAT pathway promotes angiogenesis by triggering angiogenic factor production, including vascular endothelial growth factor and matrix metalloproteases [5,6,7]. This pathway is involved in the induction or prevention of apoptosis [4].

Seven STAT proteins have been discovered and described [5,6,7]. STAT1 is activated in response to a large number of ligands and appears to be essential to the responsiveness to IFN-α and IFN-γ. Moreover, STAT1 is inappropriately activated in many tumors. The phosphorylation of STAT1 on Tyr 701 leads to STAT1 dimerization, nuclear translocation and DNA binding [8]. As the phosphorylation of STAT1 at Tyr 701 is essential for dimerization and DNA binding, it is an important marker of STAT1 activity.

STAT2 is rapidly activated by phosphorylation at Tyr 690 in response to IFNs and plays an important role in antiviral responses and carcinogenesis [9]. STAT2 does not form homodimers, but activated STAT2 forms a heterodimer with STAT1 and translocates to the nucleus [10].

STAT3 is a crucial signaling molecule for many cytokines and growth factor receptors. This molecule is constitutively activated in a number of human and animal tumors and is activated by a variety of cytokines, including interleukins, EPO and many others [11]. The phosphorylation of STAT3 at Tyr 705 induces its dimerization, nuclear translocation and DNA binding. 

STAT4 is activated by phosphorylation at Tyr693 in response to IL-12 and IL-23, and can contribute to autoimmune responses [12].

STAT5a and STAT5b are activated in response to a wide variety of ligands, including IL-2, growth hormone, prolactin, and granulocyte-macrophage colony-stimulating factor (GM-CSF). Phosphorylation at Tyr694 is essential for STAT 5 activation. STAT5 is constitutively active in some leukemia cell types [13]. STAT5a and STA5b are regulated and activated independently in various cell types. STAT5a, upon activation with interferon, is mainly activated in U-937 cells, while in contrast STAT5b is activated in HeLa cells [14].

Phosphorylation on Tyr 641 is obligatory for STAT6 activation in response to IL-4 and IL-13 [15]. IFN-α is able to activate the STAT6 protein, which, together with STAT5, forms a transcriptionally active heterodimer in Burkitt’s lymphoma Daudi cells [16]. In addition, it was shown that STAT6 forms a complex with STAT2 and p48 in B cells [17].

Osteoblastic cells play a crucial role in the osseointegration of dental implants. However, little is known regarding the possible impact of titanium and zirconia implants on the surrounding tissue, especially the activation of the JAK–STAT pathway [3]. Due to the variety of biological functions of the JAK–STAT pathway, alterations may have a wide range of effects [18,19,20]. Many research groups have investigated the role of the JAK–STAT pathway in various cells, including MG-63; however, little is known about the activation of this pathway when the cells are attached onto zirconia and titanium surfaces.

The aim of the study was to investigate whether zirconia or titanium discs influence the activation of the JAK–STAT pathway in MG-63 cells following incubation with interferon-alpha (IFN-α), interferon-gamma (IFN-γ), interleukin 4 (IL-4) and interleukin 6 (IL-6). To our knowledge, this is the first in vitro study that focuses on the influence of titanium and zirconia on the JAK–STAT pathway.

## 2. Materials and Methods

### 2.1. Materials and Specimens

Two bespoke discs (15 × 1.5 mm) were used in this study: a machined titanium disc (T) with a polished surface obtained through a grinding process, which corresponds to the clinically standardized polished and smooth regions typically used for tissue-level dental implants, and a polished zirconia disc (Z), which corresponds to the polished part of the Straumann^®^ (Basel, Switzerland) PURE CERAMIC MONOTYPE implant. All tested control groups were manufactured and provided by Institute Straumann AG, Basel, Switzerland. A detailed protocol of disc preparation and their characteristics in terms of roughness and wettability is described in previous studies [21,22].

DMEM medium for cell culture, fetal bovine serum, Trypsin-EDTA, supplements and basic laboratory chemicals were purchased from Merck (Buchs, Switzerland). Human recombinant IFN-alpha (IFN-α), IFN-gamma (IFN-γ), interleukin (IL)-6 and IL-4 were purchased from R&D Systems (Minneapolis, MN, USA). Primary rabbit polyclonal antibodies against phosphorylated human STAT proteins for Western blot and immunofluorescence experiments as well as antibodies recognizing total STAT protein independent from phosphorylation were from Cell Signaling (Danvers, MA, USA). The recognized phosphorylated tyrosines residues on the respective STAT proteins can be summarized as follows: epitope Y701 on STAT1, epitope Y705 on STAT3, and epitope Y641 on STAT6. The monoclonal antibodies against human vinculin, and the secondary antibodies conjugated with Alexa Fluor 488 (goat anti-mouse) and Alexa Fluor 555 (goat anti-rabbit), for the immunofluorescence experiments were from Merck (Buchs, Switzerland). The secondary antibodies for Western blot analysis were goat-anti rabbit conjugated with horseradish peroxidase (Thermo Fisher, Waltham, MA, USA). The antibodies for flow cytometry used in this study were Alexa Fluor 488 mouse anti-human phospho-STAT1 (epitope Y701), anti-phospho-STAT3 (epitope Y705), anti-phospho-STAT6 (epitope Y641), FITC-conjugated mouse anti-human MHC class II (HLA-DR and HLA-DQ), as well as isotype-matched IgG for flow cytometry experiments, which were purchased from BD Biosciences (San Jose, CA, USA). The Peridinin–Chlorophyll Protein Complex (PerCP)-conjugated mouse anti-human monoclonal antibodies against major histocompatibility complex (MHC class I) human leukocyte antigen-A, B, C (HLA-A, B, C) were from BioLegend (San Diego, CA, USA).

### 2.2. Cell Culture

The human osteosarcoma cell line MG-63 (CRL-1427) and human retinal pigment epithelial cell line ARPE-19 (CRL-2302), which were used as control, were purchased from the American Type Culture Collection (ATCC, Manassas, VA, USA). The cell lines used were accompanied by identification test certificates and were grown according to corresponding tissue culture collection protocols. The ARPE-19 cells were grown in Dulbecco’s minimal essential medium (DMEM)/F12 and MG-63 were grown in DMEM supplemented with 10% Fetal calf serum (FCS) and 1% Penicillin-Streptomycin Solution at 37 °C, 5% CO_2_ and 100% humidity. The FCS, DMEM and Trypsin EDTA solution were from Bioconcept (Allschwil, Switzerland). All other chemicals employed in this study were from Merck (Buchs, Switzerland) and of the highest grade of purity. All the cell culture experiments were performed in TPP plastic ware (Trasadingen, Switzerland).

### 2.3. Western Blot

The Western blot analysis of phospho-STAT and unphosphorylated STAT protein was performed as described before [23]. Briefly, MG-63 cells were cultivated on T or Z discs for 72 h. Cytokine-treated (10 ng/mL for 20 min at 37 °C) or untreated cells (negative control) were lysed in lysis buffer (50 mM Tris–HCl, 5 mM EDTA, 150 mM NaCl, 0.5% Nonidet-40, 1 mM PMSF, 10 µM Sodium vanadate and protein inhibitors aprotinin, leupeptin and pepstatin (1 µg/mL each), pH 8.0) on ice for 30 min. Afterwards, a centrifugation for 5 min at full speed was performed; 40 µg total protein was mixed with 4× NuPAGE LDS loading buffer from Invitrogen (Carlsbad, CA, USA) and resolved on NuPAGENovex 4–12% Bis-Tris gels. The proteins were transferred to a nitrocellulose membrane using 1x Novex Tris-Glycine Transfer buffer (Invitrogen, Waltham, MA, USA) according to the manufacturer’s instructions. Nonspecific binding sites were blocked with 5% milk in TBST (120 mM Tris–HCl, pH 7.4, 150 mM NaCl, and 0.05% Tween 20) for 1 h at room temperature. Target proteins were detected using specific STAT antibodies recognizing phospho-STAT proteins or total STAT proteins, as is outlined above. The membranes were washed three times and incubated with secondary antibodies conjugated with horseradish peroxidase immune complexes, and were visualized using the enhanced chemiluminescence system (Bio-Rad, Hercules, CA, USA). Data are representative of three independent experiments with nearly identical results.

### 2.4. Immunofluorescence Staining

MG-63 cells were cultured on T or Z discs for 72 h in 24-well tissue culture dishes. In addition, the MG-63 cells were cultivated on 12 mm cover glasses. The cells were left untreated (negative control) or incubated with appropriate cytokines for 20 min at 37 °C. Then cells were fixed in ice cold methanol/acetone (1:1) for 20 min, washed with PBS and further incubated with 10% goat serum in PBS for one hour at room temperature. Rabbit anti-human phospho-STAT and mouse anti-human vinculin antibodies were used as primary antibodies (dilution 1:100). Goat-anti-mouse Alexa Fluor 488 and goat anti-rabbit Alexa Fluor 555 were used as secondary antibodies (dilution 1:1000). The images were collected and analyzed on an Olympus BX-51 microscope with 20× objective using proprietary software as described before [24].

### 2.5. Analysis of Activated STAT Proteins by Flow Cytometry

MG-63 cells were incubated on discs for 72 h, washed with PBS and incubated with 10 ng/mL cytokine for 20 min at 37 °C, harvested, washed and fixed with 2% PFA for 30 min at RT and permeabilized with 0.1% TX-100 for 5 min at RT, as was previously described [21]. The MG cells cultured on wells without discs were used for comparison. Then the cells were stained o/n at 4 °C with Alexa Fluor 488-conjugated anti-phospho-STAT1, anti-phospho-STAT3, or anti-phospho-STAT6 antibodies, as is indicated in the figure legends and collected on an FACS Calibur cytometer with CellQuest Pro software (Becton Dickinson, Franklin Lakes, NJ, USA). Unstained cells or cells stained with isotype-matched IgG antibodies served as a negative control. All experiments were performed three times. The mean fluorescence intensities were quantitatively analyzed (20,000 events in each variant). The images were collected on a Becton Dickinson FACS Calibur using Cell Quest Pro Software.

### 2.6. HLA Modulation Assay

The surface expressions of MHC class I and class II were monitored by flow cytometry, using a Peridinin–Chlorophyll Protein Complex (PerCP)-labeled mouse anti-human monoclonal antibody for HLA-A, B and C heavy chains, and fluorescein isothiocyanate (FITC)-conjugated mouse anti-human antibodies against MHC class II (HLA-DR, HLA-DQ) or control, isotype-matched antibodies, in cells cultured for 48 h in the presence or absence of IFN-α and IFN-γ, as was described previously [22]. The mean fluorescence intensity of the stained cells was measured and analyzed using an FACS Calibur analyzer and the CellQuest Pro Software.

### 2.7. MTT Assay

Thirty thousand MG-63 cells were cultivated on discs in 24-well cell culture plates; 72 h later, the MTT 0.1 mg/mL was added, and the cells were incubated for a further 4 h. The reaction was stopped by adding 125 µL of DMSO. The supernatants were harvested, and the optical density was measured at 590 nm, as was described previously [25]. Three independent experiments were performed in triplicates.

### 2.8. Statistical Analysis

Data were collected in an Excel sheet (Microsoft Corporation, Richmond, CA, USA) for descriptive analysis. Student’s *t*-test was applied (IBM^®^, SPSS^®^ Statistics software Version 26.0 (IBM Corp., Armonk, NY, USA)) to assess statistically significant differences between the samples in the experiments conducted. Statistical probabilities (*p*) were expressed as * when *p* < 0.05; ** when *p* < 0.01; and *** when *p* <0.001.

## 3. Results

### 3.1. Western Blot Analysis of STAT Proteins

**Figure 1:**
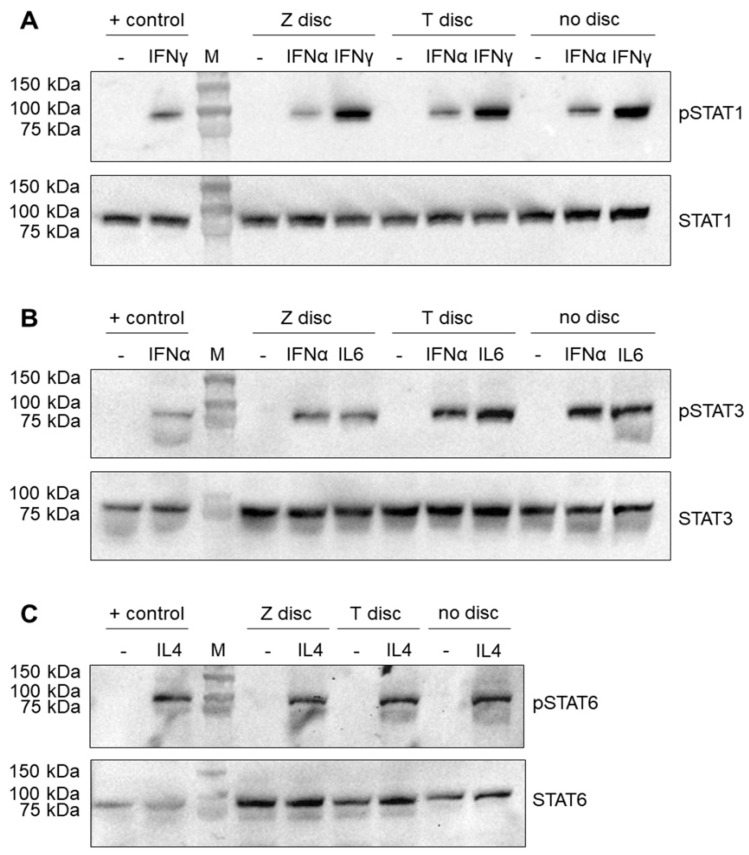
Western blot analysis of STAT proteins. The MG-63 cells were incubated for 72 h on T or Z discs or in wells without any discs. The cells were left untreated (negative control) or treated with an appropriate cytokine for 20 min. (**A**) Stimulation with IFN-α or IFN-γ. The membranes after the transfer of separated proteins were probed with anti-phospho-STAT1 (upper part) and anti-STAT1 antibodies (lower part); (**B**) stimulation with IFN-α or IL-6. Phospho-STAT3 and STAT3 proteins were detected using phospho-STAT3 and STAT3 antibodies, respectively (upper and lower part); (**C**) stimulation with IL-4 and detection of phospho-STAT6 and STAT6 proteins (upper and lower part). The protein lysates of ARPE-19 cells were used as a positive control for the detection of phosphorylated and unphosphorylated STAT proteins.

The MG-63 cells were cultivated on discs for 72 h, and then treated with appropriate cytokine or left untreated as a negative control. As shown in Figure 1A, a strong activation of the phopsho-STAT1 protein was observed in MG-63 cells after stimulation with IFN-γ. The band of phospho-STAT1 after stimulation with IFN-α was much weaker (Figure 1A, upper part). The Western blot results did not show any differences between the T and Z discs, or in a sample that was cultured without any discs. The activation of STAT1 was specific, and the untreated control samples do not show any traces of phospho-STAT1. It should be mentioned that the phospho-STAT1 antibody detects endogenous levels of STAT1 only when phosphorylated at Tyr 701. It does not cross-react with corresponding phospho-tyrosines of other STAT proteins. The membranes were also probed with a STAT1 antibody, which detects endogenous levels of total STAT1 independent of phosphorylation (Figure 1A, lower part). A strong STAT1 was observed in all samples (both stimulated and unstimulated). As a positive control for Western blot experiments, the lysates of ARPE-19 cells, untreated and treated with various cytokines [26], were used in this study.

MG-63 cells were also stimulated with IL-6 and IFN-α to detect the phospho-STAT3 protein. As shown in Figure 1B (upper panel), after stimulation with both IL-6 and IFN-α, a strong phospho-STAT3 band (the samples that were left untreated did not show any traces of phospho-STAT3) was observed. In contrast, when the membranes were probed with STAT3 antibody, which recognizes total STAT3 independent of phosphorylation, the band corresponding to the STAT3 protein was observed in all samples (Figure 1B, lower panel).

Finally, the MG-63 cells after 72 h of incubation on T or Z discs were stimulated for 20 min with IL-4. As shown in Figure 1C (upper panel), this stimulation led to the activation of the phospho-STAT6 protein, which was detected by the specific anti-phospho-STAT6 antibody. The untreated samples did not show any traces of activated STAT6. When a membrane was probed with the anti-STAT6 antibody, a strong STAT6 band was visible in all samples (stimulated and unstimulated with IL-4) (Figure 1C, lower panel).

### 3.2. Immunofluorescence Analysis of STAT Proteins

**Figure 2:**
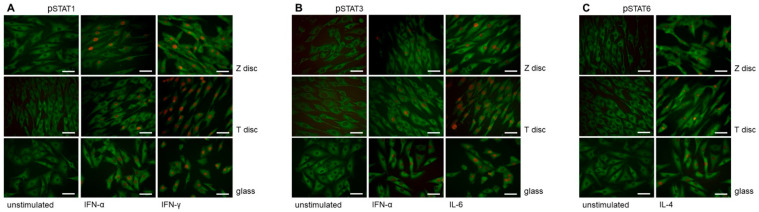
Immunofluorescence analysis of phospho-STAT proteins in MG-63 cells cultivated on discs (T and Z) or cover glasses for 72 h. (**A**) Phospho-STAT1 staining. The MG-63 cells were untreated (left panel) or treated with IFN-α (middle panel) or IFN-γ (right panel). Untreated and IFN-α/IFN-γ-treated cells were stained with anti-phospho-STAT1 and anti-vinculin antibodies. When the MG-63 cells were stimulated with IFN-α or IFN-γ, an intense nuclear immunostaining of phospho-STAT1 was observed (middle and right panels). The cytoplasm vinculin staining is shown in green. (**B**) Phospho-STAT3 staining. The MG-63 cells were untreated (left panel) or treated with IFN-α (middle panel) or IL-6 (right panel). All cells were incubated with anti-phospho-STAT3 antibodies (red) and anti-vinculin (green). In MG-63 cells stimulated with IFN-α or IL-6 an intense nuclear immunostaining of phospho-STAT3 was observed (right panel). (**C**) Phospho-STAT6 staining. The MG-63 cells were left untreated or treated with IL-4 (right panel). The cells were incubated with anti-phospho-STAT6 (red) and anti-vinculin (green) antibodies. In MG-63 cells stimulated with IL-4, an intense nuclear immunostaining of phospho-STAT6 was observed (right panel). In all images magnification 40×, scale bar—50 nm.

**Figure 3:**
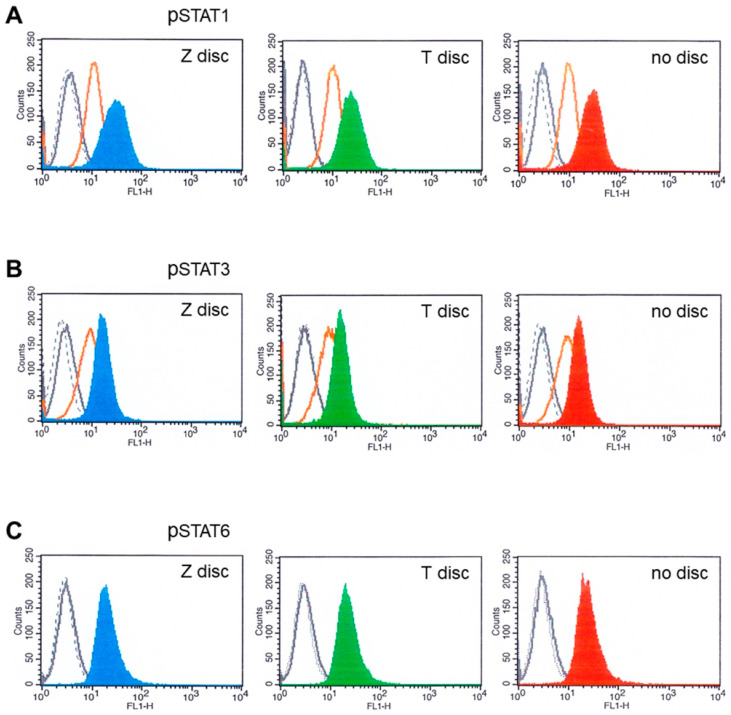
Flow cytometry analysis of MG-63 cells cultivated for 72 h on T or Z and then stimulated with IFN-α or IFN-γ, as well as with IL-6 and IL-4. (**A**) Phospho-STAT1 staining. The fluorescence intensity of MG-63 cells on Z disc (left pictures), on T discs (middle pictures) and without discs (right pictures). MG-63 cells stained with isotype-matched control antibodies (negative control)—black dotted histograms; unstimulated cells—grey histograms (sometimes they completely overlap with the peaks of isotype control). MG-63 cells stimulated with IFN-α (orange histograms) and stimulated with IFN-γ on Z disc (blue histogram, on T discs—green; without discs—red histogram). (**B**) Stimulation with IL-6 and analysis of phospho-STAT3. Black dotted histogram—isotype matched control. Unstimulated cells—grey histograms (almost overlap with isotype control histograms), MG-63 cells stimulated with IFN-α (orange histograms) and MG-63 cells stimulated with IL-6 on Z disc (blue histogram), on T discs (green) and without discs (red histogram). (**C**) Stimulation with IL-4 and analysis of phospho-STAT6. Black dotted histogram—isotype matched control. Unstimulated cells—grey histograms (almost overlap with isotype control histograms), MG-63 cells stimulated with IL-4 on Z disc (blue histogram), on T discs (green) and without discs (red histogram).

The nuclear translocation of STAT1, STAT3 and STAT6 proteins after incubation of MG-63 cells on T or Z discs for 72 h and stimulation with the appropriate cytokine is shown in Figure 2. For STAT1 activation, the MG-63 cells were treated with IFN-α or IFN-γ (Figure 2A); for STAT3 activation, the cells were treated with IFN-α and IL-6 (Figure 2B), and STAT6 nuclear translocation after treatment with IL-4 is shown in Figure 2C. The MG-63 cells are shown untreated (left panel) or after short time (20 min) of treatment with IFN-α (middle panel) or IFN-γ (right panel, Figure 2A), with nuclear translocation observed using specific anti-phospho-STAT1 antibody. The cytoplasm was stained with anti-vinculin antibodies. In untreated cells, only vinculin staining is visible, whereas in interferon-treated cells the nuclear localization of the STAT1 protein could be visualized. As shown in Figure 2B, a strong STAT3 translocation was observed in MG-63 cells upon stimulation with IL-6 (Figure 2B, right panel), and the STAT3 staining after stimulation with IFN-α (Figure 2B, middle panel) was weaker. A strong nuclear translocation of phospho-STAT6 after stimulation with IL-4 was observed in MG-63 cells cultivated on T and Z discs, as well as in cells cultured on cover glasses (Figure 3C, right panel).

### 3.3. Flow Cytometry Analysis of STAT Proteins

In the next set of experiments, the expression of activated STAT proteins was analyzed by flow cytometry. The MG-63 cells were incubated on T and Z discs, or on plastic ware without discs, for 72 h. The cells were then stimulated for 20 min with an appropriate cytokine and analyzed for the expression of phospho-STAT1, phospho-STAT3 and phospho-STAT6 proteins.

The expression of phosphorylated STAT proteins in MG-63 cells after stimulation with 10 ng/mL of IFN-α, or IFN-γ, as well as with IL-6 and IL-4, is shown in Figure 3.

As presented in Figure 3A, the MG-63 cells expressed high amounts of STAT1 after stimulation with IFN-γ (Figure 3: blue, green and red histograms), whereas after stimulation with IFN-α, the STAT1 expression was less strong (orange histograms). Similar results were observed when the MG-63 cells were stimulated with IFN-α and IL-6 and probed with phospho-STAT3 antibodies (Figure 3B). We also checked if the IL-4 activates STAT6 in flow cytometry. As shown in Figure 3C, IL-4 activated STAT6 when the cells were cultivated on discs, as well as in a sample without discs (Figure 3C, red histogram). No significant differences in fluorescence intensities were detected between the samples from T or Z discs, or in samples without discs.

### 3.4. MHC Modulation

**Figure 4:**
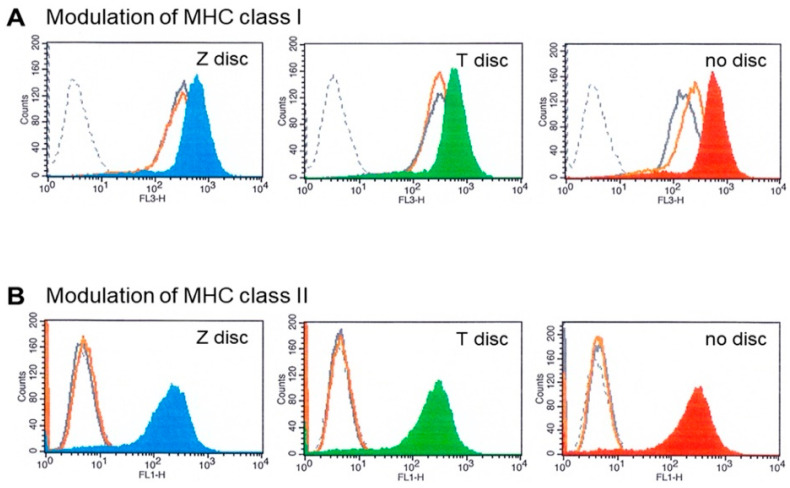
(**A**) MHC class I modulation. Flow cytometry analysis of MG-63 cells cultivated for 72 h on T or Z discs and then stimulated with IFN-α or IFN-γ for 48 h. The MG-63 cells express high amounts of MHC class I (grey histograms), after stimulation with IFN-γ the overexpression of MHC class I was observed in the MG-63 cells (on Z disc—blue histogram; on T discs—green; in a sample without discs—red histogram). Stimulation with IFN-α did not change the MHC class I expression (orange histograms). The black dotted histograms represent isotype-matched negative controls. (**B**) MHC class II modulation. Flow cytometry analysis of MG-63 cells cultivated for 72 h on Z or T discs and then stimulated with IFN-α and IFN-γ for 48 h. The expression of MHC class II was analyzed. After stimulation with IFN-γ the MG-63 cells overexpressed MHC class II proteins (on Z disc—blue histogram; on T discs—green; without discs—red histogram). Stimulation with IFN-α did not influence the MHC class II expression (orange histograms), which overlap with the histograms of unstimulated cells (grey histograms) and with histograms of isotype control (black dotted histograms).

The effects of IFN-α and IFN-γ on MHC class I and MHC class II expression on MG-63 cells were tested by flow cytometry. Untreated MG-63 cells express high levels of MHC class I (Figure 4A,B—grey histograms). Black dotted histograms show isotype-matched negative controls. When the MG-63 cells were treated with 10 ng/mL of IFN-γ for 48 h, the upregulation of MHC class I expression was observed (Figure 4A blue and green histograms) on Z and T discs, respectively, as well as in a sample without any discs (right histogram). In contrast, IFN-α treatment did not have any effect on the overexpression of MHC I (the histograms of unstimulated cells (grey histograms) and IFN-α treated cells (Figure 4A, orange histograms) almost overlap). The MG-63 cells were treated with IFN-α the same way as with IFN-γ (for 48 h). Expression of MHC class II was also examined. The results are shown in Figure 4B. The stimulation with IFN-α (orange histograms) did not alter the expression profile of MHC class II molecule (Figure 4B). In contrast, 48 h incubation with IFN-γ led to the upregulation of MHC class II expression in all examined samples.

### 3.5. MTT Assay

**Figure 5:**
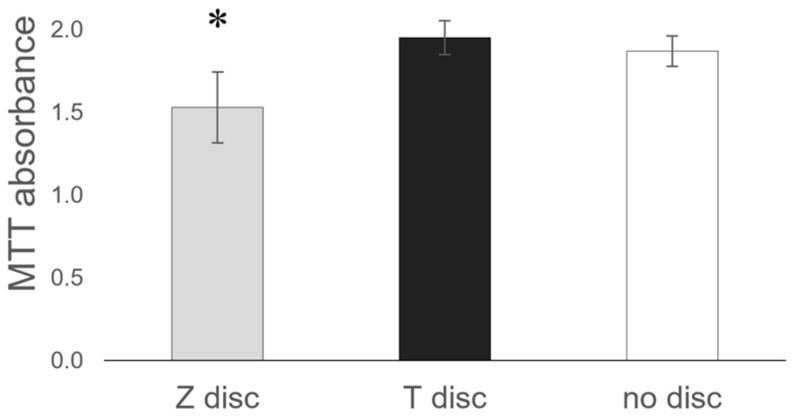
Comparison of the Z and T discs in MTT assay. MG-63 cells were incubated on discs for 72 h, then the MTT assay was performed. Values represent mean ± SD, * *p* < 0.05. Three independent experiments were performed in triplicates.

Incubation of MG-63 cells on the surface of T or Z discs did not influence their cellular metabolic activity. As shown in Figure 5, no significant differences in MTT absorbance between T discs and samples without discs (used as a control) were observed. In contrast, on Z discs, the MG-63 cells showed a slightly decreased metabolic activity.

## 4. Discussion

Zirconia implants are a promising alternative to the well-established titanium implants. and might have several biological benefits, including the inhibition of bacterial adhesion, enhanced soft tissue adhesion and improved osseointegration [27,28].

However, bone cells and the remodeling process are influenced by cytokines, which act as a strong stimulatory factor of bone formation. To increase osseointegration, a superior activation or upregulation of the JAK–STAT pathway might be beneficial.

Western blot data have shown that three STAT proteins (STAT1, STAT3 and STAT6) could be activated in MG-63 cells after stimulation with IFN-α and IFN-γ, as well as with IL-6 and IL-4. Antibodies, which recognize total STAT protein independent of phosphorylation, have shown that all samples express STAT protein. The expression of corresponding phospho-STAT proteins were detected only after stimulation with an appropriate cytokine. The strongest response was shown to IFN-γ. The surfaces (titanium or zirconia) did not have any influence, and the STAT activation patterns were very similar. Flow cytometry data confirm these findings. All examined STAT proteins could be activated in MG-63 cells independent of the surface.

Cellular development, growth, and homeostasis are all mediated by the JAK–STAT pathway. Interferons exhibit their antiviral properties through the induction of MHC molecules’ expression on the surface of immunocompetent cells, which in turn enhances antigen presentation to CD8-positive cytotoxic T cells. The transcriptional activity of phosphorylated STAT dimers in response to IFN-γ within 48 h were reflected in a significant upregulation of both MHC class I and MHC class II proteins in MG-63 cells. This upregulation was observed in all samples tested. The histograms obtained from MG-63 cells after incubation on T or Z discs showed similar results. The MHC class II modulation was also observed in all samples after stimulation with IFN-γ. In contrast, stimulation with IFN-α did not have any effect on MHC class I and MHC class II modulation on all surfaces.

Upon stimulation with cytokines, STAT proteins were translocated to the nucleus, as demonstrated by immunofluorescence experiments, indicating necessary phosphorylation at specific tyrosine residues and their dimerization. Here, again, the strongest response was observed after stimulation with IFN-γ. IL-6 and IL-4 activated STAT3 and STAT6, respectively.

The obtained data demonstrate that the JAK–STAT signaling pathway is unimpaired, and the use of neither a zirconia nor a titanium surface changed the abilities of MG-63 cells to respond to cytokines by activating the members of the JAK–STAT family. As a control, the MG-63 cells were cultured in tissue culture plastic without any discs. All groups (Z and Z discs, as well as in samples without discs) revealed very similar results. Our major findings are summarized in Table 1.

As shown in Table 1, the individual activation patterns were cytokine-specific. Our experimental data have shown that IFN-γ induced a very strong activation of STAT1 in all tested assays (Western blot, immunofluorescence and flow cytometry). IFN-α induced a less strong activation of STAT proteins (both STAT1 and STAT3) compared to IFN-γ and IL-6. The activation of STAT1 after incubation with IFN-α was relatively strong in Western blot, but weak in flow cytometry. It is important to use at least two methods to characterize the activation of STAT proteins after stimulation with a cytokine. In sum, our data show that IFN-α activated STAT1 and STAT3, IFN-γ activated STAT1, and IL-6 and IL-4 activated STAT3 and STAT6, respectively.

The JAK–STAT pathway regulates bone homeostasis and is involved in the proliferation and differentiation of various cell types, including osteoblasts and osteoclasts [29,30]. Bellido et al. demonstrated that IL-6-type cytokines promote osteoblast differentiation, which is mediated by the activation of the JAK–STAT signaling pathway [31]. De Souza et al. have shown that the effect of oncostatin on osteoclastogenesis is mediated by the JAK–STAT/MAPK pathway [32]. As previously shown, the activation of the JAK–STAT signaling pathway strongly correlates with osteogenic differentiation of human bone marrow mesenchymal stem cells. Furthermore, immunomodulatory responses enhance osseointegration [33]. Sanpaolo et al. have provided a detailed overview of the involvement of the JAK–STAT pathway in bone remodeling, and have shown that further studies are needed to characterize the molecular mechanisms underlying these bone process [34]. Likewise, over a 12-week period, Depprich et al. evaluated the osseointegration of zirconia implants versus titanium implants. Their findings suggest that zirconia implants with altered surfaces achieve osseointegration equivalent to titanium implants [35], which is in line with the data shown in this study. Rausch et al. have shown that the behaviour of primary human gingival fibroblasts is primarily affected by surface structure, whereas no apparent advantage of zirconium over titanium has been observed [21]. Meanwhile, Sun et al. studied the effect of curcumin on osteosarcoma cells, hypothesizing that the inactivation of the JAK–STAT pathway has an impact on MG-63 cells [36]. Annamalai et al. reported that the anticancer effects of β-caryophyllene (BCP) promote apoptosis and inflammation via reactive oxygen species as a result of the activation of the JAK–STAT signaling pathway in MG-63 cells [37]. These findings suggest that the JAK–STAT pathway can be affected by various factors. Additionally, the activation of STAT1 and STAT3 proteins may trigger the activation of many genes involved in immunosuppression and the induction of tolerance [38,39,40].

The present study demonstrates that STAT1, STAT3 and STAT6 are activated in MG-63 cells when cultivated on T or Z discs. However, the JAK–STAT pathway is not the only pathway that could be activated in cells upon incubation on the T and Z discs in response to human cytokines and growth factors. Therefore, other signaling pathways (e.g., Nuclear Factor kappa B) must be investigated in detail, as Pantouli et al. discussed the role of the P38 MAPK pathway in the induction of osteoprotegerin synthesis by MG-63 cells [41]. Further studies are required to understand in detail the molecular mechanisms underlying the bone remodeling process and interactions between the immune and bone systems.

## 5. Conclusions

In the current study, JAK–STAT pathway activation in the MG-63 cells was demonstrated using Western blot, flow cytometry and immunofluorescence methods. Upon stimulation with various cytokines STAT1, STAT3 and STAT6 were activated in the MG-63 cells when they were cultured on T and Z discs. Notably, the individual STAT activation patterns were cytokine-specific.

The JAK–STAT pathway could be activated in osteoblast-like MG-63 cells on zirconia in response to interferons and cytokines, and thus represents an alternative to titanium implants.

## Figures and Tables

**Table 1:** Summary of cytokine specific activation patterns in MG63 cells.

Cytokine	Protein	WB	IF	FC
IFN-α	STAT1	++	++	++
IFN-γ	STAT1	+++	+++	+++
IFN-α	STAT3	++	++	+
IL-6	STAT3	+++	++	++
IL-4	STAT6	++	++	++

Notes: +++ = strong expression; ++ = moderate expression; + = weak expression. Abbreviations: WB—western blot, IF—immunofluorescence staining, FC—flow cytometry.

## Data Availability

Not applicable.
